# RecN spatially and temporally controls RecA-mediated repair of DNA double-strand breaks

**DOI:** 10.1016/j.jbc.2023.105466

**Published:** 2023-11-17

**Authors:** Shunsuke Noda, Genki Akanuma, Kenji Keyamura, Takashi Hishida

**Affiliations:** Department of Molecular Biology, Graduate School of Science, Gakushuin University, Tokyo, Japan

**Keywords:** DNA double-strand breaks, DNA homologous recombination, DNA repair, *Escherichia coli*, nucleoids

## Abstract

RecN, a bacterial structural maintenance of chromosomes–like protein, plays an important role in maintaining genomic integrity by facilitating the repair of DNA double-strand breaks (DSBs). However, how RecN-dependent chromosome dynamics are integrated with DSB repair remains unclear. Here, we investigated the dynamics of RecN in response to DNA damage by inducing RecN from the *P*_*BAD*_ promoter at different time points. We found that mitomycin C (MMC)-treated Δ*recN* cells exhibited nucleoid fragmentation and reduced cell survival; however, when RecN was induced with arabinose in MMC-exposed Δ*recN* cells, it increased a level of cell viability to similar extent as WT cells. Furthermore, in MMC-treated Δ*recN* cells, arabinose-induced RecN colocalized with RecA in nucleoid gaps between fragmented nucleoids and restored normal nucleoid structures. These results suggest that the aberrant nucleoid structures observed in MMC-treated Δ*recN* cells do not represent catastrophic chromosome disruption but rather an interruption of the RecA-mediated process. Thus, RecN can resume DSB repair by stimulating RecA-mediated homologous recombination, even when chromosome integrity is compromised. Our data demonstrate that RecA-mediated presynapsis and synapsis are spatiotemporally separable, wherein RecN is involved in facilitating both processes presumably by orchestrating the dynamics of both RecA and chromosomes, highlighting the essential role of RecN in the repair of DSBs.

DNA double-strand breaks (DSBs) pose a major threat to genome stability and cell survival because they compromise the structural integrity of chromosomes (1, 2). If left unrepaired or repaired incorrectly, DSBs can cause deleterious genetic alterations such as chromosomal rearrangements and chromosome loss, which are linked to cell death and cancer. Homologous recombination (HR) is an evolutionarily conserved mechanism that plays a crucial role in the faithful repair of DSBs using intact dsDNA molecules as a template (2, 3, 4). In *Escherichia coli*, the DSB end is recognized by RecBCD, and its helicase and nuclease activities process the DSB to create 3′-ssDNA (5, 6). RecA binds to the resulting ssDNA and forms a nucleoprotein filament. Once the filament forms, RecA searches for homologous DNA and promotes ssDNA invasion into homologous duplex DNA to initiate strand exchange (7, 8, 9, 10, 11). The invading 3′-ssDNA then initiates DNA repair synthesis, leading to the formation of a synaptic complex. Finally, structure-specific endonucleases and/or helicases (*e.g.*, RuvABC and RecG) resolve the joint molecules to generate the recombination products (12, 13, 14, 15). In such multistep reactions, it is believed that controlling the dynamic behavior between broken DNA and homologous donor strands plays an important role in facilitating RecA-mediated homology search and subsequent reactions, but little is known about how chromosome dynamics is coordinated with DSB repair.

Structural maintenance of chromosomes (SMC) family of proteins is conserved in prokaryotes and eukaryotes and play important roles in chromosome dynamics, including chromosome cohesion and condensation, as well as DNA repair (16, 17). At least three SMC-like proteins, MukB, SbcC, and RecN, have been identified in *E. coli* (18). MukB forms a condensin complex with two non-SMC proteins, MukE and MukF (19, 20, 21). SbcC forms a complex with the SbcD nuclease, which has a similar structure to the eukaryotic Rad50–Mre11 (22, 23), suggesting that it contributes to the generation and/or processing of DNA ends through the endonuclease/exonuclease activity of SbcD. The highly conserved bacterial RecN protein shares structural features with other SMC proteins, although its coiled-coil domain is much shorter (24). The *E. coli recN* gene has three SOS boxes in its promoter region (25, 26), and RecN protein is selectively degraded by ClpXP protease *via* the recognition of short signals in its C terminus (27, 28, 29). Thus, RecN expression is strictly limited to cells with DNA damage, indicating that it has a specific role in the DNA damage response.

*E. coli recN* mutants are highly sensitive to ionizing radiation, I-SceI cleavage, and mitomycin C (MMC) (30, 31) and exhibit an abnormal morphology in the presence of MMC characterized by highly elongated cells with short, diffuse, and *oriC*-lacking nucleoids (32). SOS-inducible RecN localizes to nucleoids in MMC-treated cells and promotes sister chromatid interactions (32, 33). Previous biochemical studies in several bacteria revealed that RecN interacts with RecA and stimulates the RecA strand exchange activity (34, 35, 36). Moreover, RecN bound to dsDNA slides along the dsDNA toward the RecA-bound ssDNA region, and ssDNA-bound RecN topologically entraps a second dsDNA molecule in an ATP-dependent manner (36). A recent live-cell imaging study of *Caulobacter crescentus* revealed that RecN regulates RecA filament dynamics during the homology search, allowing recombination between sites of homology on distant chromatids (37). Together, these results suggest that RecN functions in the RecA-mediated synaptic complex formation during DSB repair.

Aberrant nucleoid shape in MMC-treated Δ*recN* cells appears to be linked to loss of viability. However, it is unclear whether this is due to catastrophic DNA degradation induced by a failure in HR-dependent DSB repair. In this study, we developed a conditional expression system utilizing the *P*_*BAD*_ promoter to control the expression of *recN* and investigated its spatial and temporal dynamics in response to DNA damage by inducing RecN at different time points. Our results show that inducing RecN in MMC-treated Δ*recN* cells restored normal nucleoid structures and cell survival equivalent to WT cells, implying that the fragmented nucleoid structure observed in Δ*recN* cells retains repair-proficient substrates for HR-dependent DSB repair. Furthermore, in MMC-treated Δ*recN* cells, arabinose-induced RecN colocalized with RecA in the nucleoid gaps between fragmented nucleoids. These and other findings highlight the structural and functional roles of RecN in HR-dependent DSB repair, which likely involves the coordination of both RecA and chromosome dynamics, thereby facilitating RecA-mediated reactions even when chromosomal integrity is compromised.

## Results

### Construction of the inducible *recN* expression system

**Figure 1:**
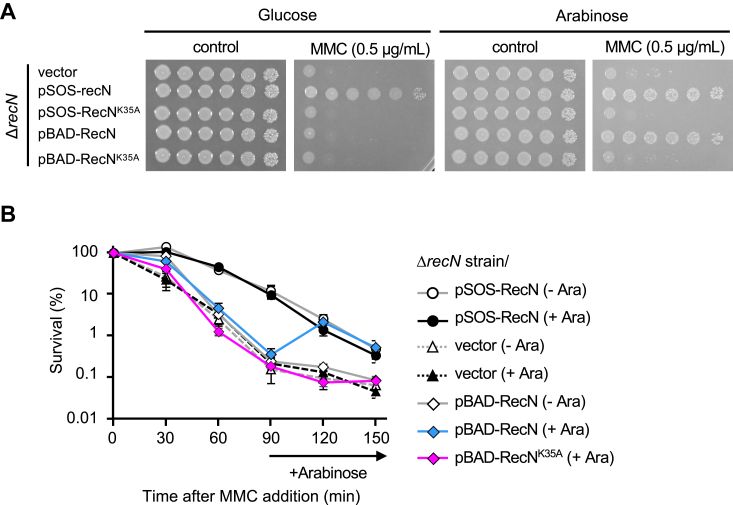
**DNA repair activity upon induction of RecN using the *P***_***BAD***_**-promoter.***A*, MMC sensitivity of Δ*recN* cells carrying the indicated plasmid. Ten-fold serial dilutions of cell cultures were spotted onto LB_Cm plates with or without MMC (0.5 μg/ml) in the presence of either glucose or arabinose. *B*, exponentially growing Δ*recN* cells carrying the indicated plasmid were exposed to MMC (0 min). Arabinose was added to the culture 90 min after MMC treatment to induce *recN* expression, followed by further incubation. Aliquots were collected at the indicated time points and plated on LB_Cm plates at appropriate dilutions. The survival rates were calculated as the number of viable cells relative to the control (0 min) samples. The data points represent the average of at least three independent experiments. Data shown are mean ± SEM. MMC, mitomycin C.

*E. coli recN* is classified as an SOS gene; its expression is tightly regulated by LexA, resulting in the rapid production of RecN in response to DNA damage (25, 28, 38, 39). However, the impact of the timing of *recN* expression on nucleoid structure and cell viability during DSB repair remains unclear. To explore this, we constructed a *recN* expression plasmid (pBAD-RecN) in which *recN* was placed under the control of the arabinose-inducible promoter (*P*_*BAD*_). In the presence of arabinose, Δ*recN* carrying pBAD-RecN (Δ*recN*/pBAD-RecN) cells showed similar sensitivity to MMC as Δ*recN*/pSOS-RecN cells, which expresses *recN* under the control of its native *P*_*SOS*_ promoter (Fig. 1*A*). However, in the absence of arabinose, Δ*recN*/pBAD-RecN cells showed high MMC sensitivity similar to Δ*recN* cells carrying the empty plasmid vector. Consistent with this, upon the addition of arabinose, RecN expression was induced in Δ*recN*/pBAD-RecN cells, and its expression pattern was comparable to that of Δ*recN*/pSOS-RecN cells (Fig. S1). Furthermore, Δ*recN*/pBAD-RecN^K35A^ cells, in which a *recN*^*K35A*^ Walker A motif mutant is expressed in response to arabinose, exhibited similar MMC sensitivity to Δ*recN* cells carrying the empty plasmid vector, even when arabinose was present (Fig. 1*A*). Hence, *P*_*BAD*_-driven RecN expression is nonleaky and tightly controlled in Δ*recN*/pBAD-RecN cells, and RecN induced in this way behaves similarly to SOS-induced RecN *in vivo*.

### Induced expression of *recN* improves cell viability and restores the integrity of nucleoid structure during MMC exposure

The viability of exponentially growing Δ*recN* cells carrying the empty plasmid vector was significantly lower after exposure to lethal doses of MMC (1.0 μg/ml) than that of Δ*recN* cells carrying pSOS-RecN (Fig. 1*B*). To investigate the influence of *recN* expression timing on the MMC sensitivity of Δ*recN* cells, Δ*recN*/pBAD-RecN cells were treated with MMC for 90 min in the absence of arabinose, followed by the induction of *recN* using arabinose. As expected, in the absence of arabinose, Δ*recN*/pBAD-RecN cells exhibited low viability equivalent to Δ*recN*/pBAD-vector cells at the 90 min mark during MMC exposure (Fig. 1*B*). Interestingly, at 30 min after arabinose addition (*i.e.*, 120 min after MMC treatment initiation), the induced expression of WT *recN*, but not *recN*^*K35A*^, increased the viability of Δ*recN* cells to a similar extent as Δ*recN*/pSOS-RecN cells (Fig. 1*B*). These findings indicate that subsequent *recN* expression enables the recovery of reduced cell survival following MMC exposure, suggesting that the loss of viability is not irreversible within the scope of our study.

**Figure 2:**
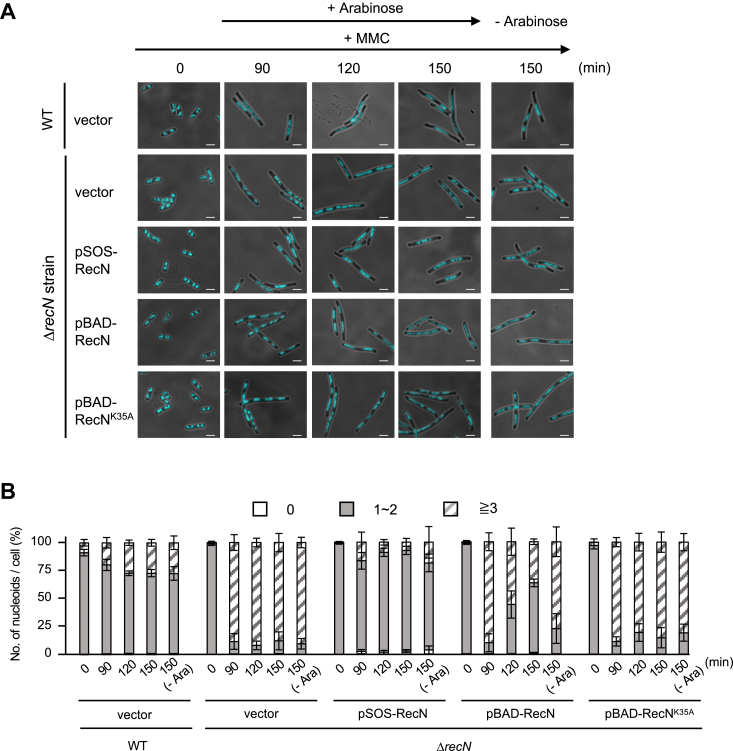
**Induced expression of *recN* increases restores nucleoid structure during MMC exposure.***A*, exponentially growing WT and Δ*recN* cells carrying the indicated plasmid were exposed to MMC (1.0 μg/ml). Arabinose was added to the culture 90 min after MMC treatment to induce *recN* expression, followed by further incubation. DAPI-stained cells were examined by fluorescence microscopy. Nucleoids are visualized as *light blue*. Scale bar represents 2.5 μm. *B*, quantification of the number of nucleoids per cell. The bar graph represents the percentages of cells with no nucleoid, 1∼2 nucleoids, and >3 nucleoids per cell. At least 100 cells were analyzed for each time point. The results represent the average of at least three independent measurements. Data shown are mean ± SD. DAPI, 4,6-diamidino-2-phenylindole; MMC, mitomycin C.

Fluorescence microscopy–based morphological analysis using 4,6-diamidino-2-phenylindole (DAPI)-stained cells revealed that MMC-treated Δ*recN* cells exhibited filamentous phenotype with short and diffuse nucleoids, a phenomenon known as nucleoid fragmentation (32). This suggests that RecN plays a crucial role in maintaining chromosome integrity during the early steps of HR-dependent DSB repair. To assess whether the induced expression of RecN can restore the integrity of nucleoid structure in MMC-treated Δ*recN* cells, we observed DAPI-stained nucleoids using fluorescence microscopy under the same conditions as described in Figure 1*B*. In the absence of MMC, Δ*recN*/pSOS-RecN and Δ*recN*/pBAD-RecN cells displayed normal morphology, with one or two nucleoids localized at the midcell or at one-fourth and three-fourth positions (Fig. 2*A*). Following a 90 min MMC treatment, Δ*recN*/pSOS-RecN cells became filamentous, but the number of nucleoids per cell remained largely unchanged, although elongation of nucleoids along the long axis was observed (Fig. 2, *A* and *B*). Conversely, Δ*recN* cells carrying either pBAD-RecN, pBAD-RecN^K35A^, or pBAD-vector exhibited more pronounced filamentation accompanied by fragmented nucleoids (90% of cells had three or more nucleoids per cell after 90 min of MMC treatment). Importantly, when arabinose was added to cell cultures at the 90 min mark during MMC treatment, the percentage of cells with normal nucleoid structures increased in Δ*recN*/pBAD-RecN cells, but not in Δ*recN*/pBAD-RecN^K35A^ cells, to a level comparable to that of Δ*recN*/pSOS-RecN cells at 60 min after arabinose induction (*i.e.*, 150 min after MMC addition) (Fig. 2, *A* and *B*). These results demonstrate that the subsequent expression of *recN* can restore chromosome integrity even after it has been compromised, as evidenced by the restoration of normal nucleoid structures. This observation is consistent with the increase in cell viability shown in Figure 1*B*.

### RecN assists RecA post presynaptic filament formation

**Figure 3:**
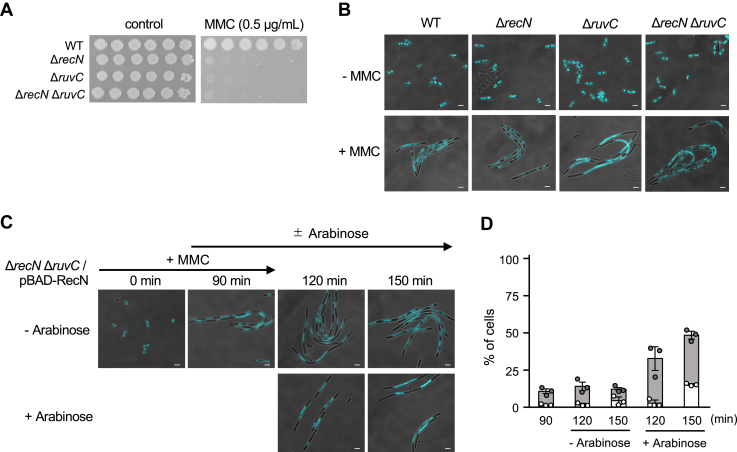
**Induction of RecN in Δ*recN* Δ*ruvC* cells leads to the accumulation of unresolved recombination intermediates.***A*, Ten-fold serial dilutions of cell cultures were spotted onto LB plates with or without MMC (0.5 μg/ml). The plates were incubated at 37 °C overnight. *B*, cell morphology and nucleoid structure of MMC-treated cells. The panels show DAPI images of cells with or without MMC (1.0 μg/ml) treatment for 90 min. Nucleoids are visualized as a *light blue color*. Scale bar represents 2.5 μm. *C*, dynamics of nucleoid structure in Δ*recN* Δ*ruvC*/pBAD-RecN cells. The cells were treated with MMC (1.0 μg/ml) for 90 min, followed by washing with M9 buffer and transfer to MMC-free medium containing arabinose. Subsequently, the cells were further incubated for 60 min (*t* = 150). Cells were collected at the indicated time points, fixed, stained, and examined using fluorescence microscopy. Scale bar represents 2.5 μm. *D*, quantitative analysis of cell morphology in (*C*). *White* and *gray* portions of bars represent the percentage of anucleate cells and cells with centrally located nucleoid aggregates, respectively. At least 100 cells were analyzed for each time point. The results represent the average of three independent measurements. Data shown are mean ± SD. DAPI, 4,6-diamidino-2-phenylindole; MMC, mitomycin C.

Previous studies have demonstrated that the RuvC protein plays a crucial role in resolving Holliday junction recombination intermediates formed by RecA (12, 13, 40). Mutations in RuvC result in increased sensitivity to DNA-damaging agents such as UV and MMC (Fig. 3*A*) (41). Microscopic observations of DAPI-stained cells further revealed that UV-treated Δ*ruvC* cells exhibited a filamentous phenotype with centrally located chromosome aggregates because of the accumulation of recombination intermediates, and subsequently produced anucleate cells (42), suggesting its involvement in the late postsynaptic stage of recombination. Similar chromosome partitioning defects were observed in MMC-treated Δ*ruvC* cells (Fig. 3*B*). Remarkably, MMC-treated Δ*recN* Δ*ruvC* double mutants displayed morphological phenotypes highly resembling those of the MMC-treated Δ*recN* single mutant (Fig. 3, *A* and *B*), suggesting that the deletion of *recN* suppresses the accumulation of recombination intermediates in Δ*ruvC* cells during MMC exposure. These results indicate that RecN functions at an earlier stage than postsynaptic phase in HR repair.

Considering that RecN potentially facilitates the process from the presynaptic to the synaptic stage of recombination, we hypothesized that inducing RecN in MMC-treated Δ*recN* Δ*ruvC* cells would result in the accumulation of recombination intermediates similar to those observed in Δ*ruvC* cells by promoting RecA-mediated reactions. To test this hypothesis, we induced RecN in Δ*recN* Δ*ruvC*/pBAD-RecN cells after release from MMC exposure and investigated its impact on nucleoid morphology. Cells were treated with MMC for 90 min and then released into MMC-free media containing arabinose. After treatment with MMC, Δ*recN* Δ*ruvC*/pBAD-RecN cells displayed a filamentous morphology with fragmented nucleoids (Fig. 3*C*). However, upon RecN induction, the number of cells with three or more nucleoids per cell decreased at 150 min (*i.e.*, 60 min after arabinose addition). Consequently, there was an increase in cells with centrally located chromosome aggregates and anucleate cells, characteristic typically associated with Δ*ruvC* mutants (Fig. 3, *C* and *D*). These findings suggest that RecN induction in this context stimulates the RecA-mediated events of the HR pathway, leading to the accumulation of unresolved recombination intermediates.

### Subcellular localization of the arabinose-induced GFP-RecN protein

*E. coli* N-terminal GFP-tagged RecN, expressed from the native SOS promoter, fully complements the MMC sensitivity of Δ*recN* cells (Fig. S2*A*) (28, 32). In contrast, in *Bacillus subtilis*, the N-terminal GFP-tagged RecN is nonfunctional, whereas the C-terminal GFP-tagged RecN behaves similarly to the WT (43). Although we are currently unable to provide a definitive explanation for this difference, it may stem from species-specific attributes of the DSB repair in *E. coli* and *B. subtilis* (44, 45). In this study, we used N-terminal GFP-tagged RecN for fluorescence microscopy analysis because it is fully functional *in vivo* (Fig. S2*A*), and the C terminus of *E. coli* RecN contains a recognition site for ClpXP, which is important for the normal RecN function (28, 29).

SOS-induced GFP-RecN forms foci on nucleoids and at the cell poles in response to MMC exposure (Fig. S2, *B* and *C*). The nucleoid localization of RecN foci requires both DNA damage and RecA, whereas cell pole RecN foci localize independently and are likely inactive RecN aggregates (32). To investigate the dynamics of GFP-RecN, we monitored its subcellular localization in Δ*recN* cells carrying the arabinose-inducible GFP-RecN plasmid (pBAD-GFP-RecN). Cells in logarithmic phase were treated with MMC for 10 min, and then immediately transferred to MMC-free medium to recover for 30 min. When arabinose was added to Δ*recN*/pBAD-GFP-RecN cultures concurrently with MMC treatment to mimic the DNA damage–induced SOS response, we observed GFP-RecN foci associated with nucleoids in 50% of cells at 30 min after release from MMC treatment (Fig. S2, *B* and *C*). Furthermore, Δ*recN*/pBAD-GFP-RecN fully complemented the repair deficiency of the Δ*recN* strain in the presence of arabinose but not in its absence (Fig. S2*A*). These results indicate that arabinose-induced RecN localizes to nucleoids, as previously observed in cells induced for the SOS response (32), when *recN* expression coincides with MMC addition to the culture.

**Figure 4:**
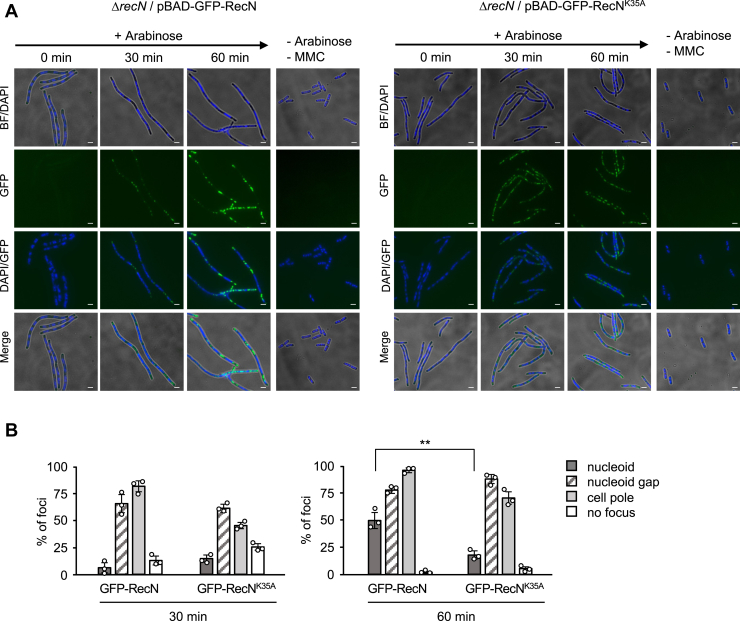
**Subcellular localization of GFP-RecN induced at different time points.***A*, nucleoid gap localization of GFP-RecN foci. Δ*recN* cells carrying either pBAD-GFP-RecN or pBAD-GFP-RecN^K35A^ were treated with MMC (1.0 μg/ml) for 90 min and released into MMC-free medium containing arabinose. Cells were fixed and stained with DAPI and analyzed by fluorescence microscopy. The panels show BF/DAPI, GFP, GFP/DAPI, and merge (GFP/DAPI/BF) images of cells after arabinose addition. Nucleoids are visualized as a *dark blue color*. Scale bar represents 2.5 μm. *B*, quantitative analysis of GFP-RecN foci in (*A*). At least 100 cells were examined for each sample. The results represent the average of three independent measurements. Data shown are mean ± SD. *p* Values were calculated by unpaired Student's *t* test; ∗∗*p* < 0.01. BF, bright field; DAPI, 4,6-diamidino-2-phenylindole; MMC, mitomycin C.

Next, we treated Δ*recN*/pBAD-GFP-RecN cells with MMC for 90 min in the absence of arabinose and then transferred them to arabinose-containing medium without MMC to induce RecN. Initially, the cells treated with MMC were highly filamentous with short diffuse nucleoids, and no GFP-RecN foci were observed (Fig. 4*A*; 0 min). However, following the induction of GFP-RecN with arabinose, multiple RecN foci rapidly appeared within the 30 min time frame, and they were primarily localized to the spaces between the nucleoids, including their edges (*i.e.*, nucleoid gaps) (Fig. 4*A*). At 60 min after GFP-RecN induction, the fluorescence intensity increased further and the number of RecN foci became more abundant (Fig. 4*A*). Some of the GFP-RecN foci were also observed within nucleoids (Fig. 4, *A* and *B*). The increase in the number of nucleoid foci at 60 min suggests the recovery of intact nucleoid structures. In addition, we observed a similar localization pattern of GFP-RecN^K35A^, 30 min after GFP-RecN^K35A^ induction (Fig. 4, *A* and *B*). However, GFP-RecN^K35A^ remained localized to nucleoid gaps at 60 min after release from MMC exposure, and no increase of nucleoid-associated foci was observed (Fig. 4, *A* and *B*). Taken together, these findings suggest that the localization of GFP-RecN to nucleoid gap is a result of its recruitment to sites of MMC-induced DSBs, and the ATPase-defective GFP-RecN^K35A^ is proficient in recruiting to sites of DNA damage but fails to recover broken nucleoid structures because of defects in HR repair.

### Time-lapse imaging of the arabinose-inducible GFP-RecN protein

**Figure 5:**
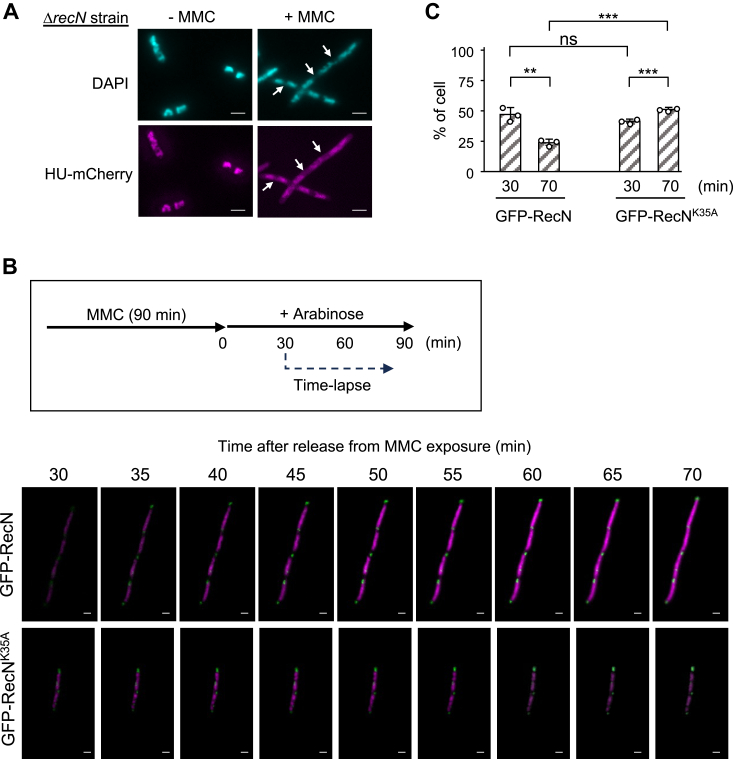
**Time-lapse fluorescence imaging of HU-mCherry and GFP-RecN.***A*, nucleoid structure of Δ*recN* cells expressing HU-mCherry (MECS123 Δ*recN*) with or without MMC (1.5 μg/ml) treatment for 90 min. Cells were fixed and stained with DAPI, and analyzed by fluorescence microscopy. *White arrows* indicate nucleoid gaps that are barely detectable by DAPI staining. Scale bar represents 2.0 μm. *B*, MECS123 Δ*recN* cells carrying the indicated plasmid were treated with MMC for 90 min and released into MMC-free medium supplemented with arabinose. Following an additional 30 min incubation, the cell culture was diluted and mounted on an agarose pad containing M9 buffer and arabinose (1% w/v). Images were acquired using a Keyence BZ-X710 microscope with a 100× oil objective at 1 min intervals. The panels show merged mCherry/GFP images of representative cells carrying either a pBAD-GFP-RecN (*upper panels*) or a pBAD-GFP-RecN^K35A^ (*lower panels*). Scale bar represents 2.0 μm. The images correspond to Movies S1 and S2. *C*, quantitative analysis of cell morphology in (*B*). The bar graph represents the percentages of cells with >3 nucleoids per cell. At least 100 cells were analyzed for each time point. The results represent the average of three independent measurements. Data shown are mean ± SD. *p* Values were calculated by unpaired Student's *t* test; ∗∗*p* < 0.01; ∗∗∗*p* < 0.001; ns, not significant (*p* > 0.05). BF, bright field; DAPI, 4,6-diamidino-2-phenylindole; MMC, mitomycin C.

To gain further insight into the dynamics of GFP-RecN in living cells, we constructed Δ*recN hapA*-mCherry strain that chromosomally expressed mCherry-tagged HU (HU-mCherry). HU is a nucleoid-associated protein that is abundantly present and binds uniformly across the genome, allowing easy visualization of nucleoids in live cells (46, 47). Upon treating Δ*recN hapA*-mCherry cells with MMC (1.5 μg/ml) for 90 min, we observed weak localization of HU-mCherry to nucleoid gaps, which were challenging to detect with DAPI staining alone (Fig. 5*A*). This observation suggests that DAPI-unstained nucleoid gaps observed in MMC-treated Δ*recN* cells represent regions with less compacted DNA structure because of DNA damage (see the Discussion section). Subsequently, we introduced pBAD-GFP-RecN into Δ*recN hapA*-mCherry cells and performed live-cell imaging to monitor the dynamics of arabinose-induced GFP-RecN. The transformants were treated with MMC for 90 min and then transferred to MMC-free media in the presence of arabinose to induce GFP-RecN. After an additional 30 min of incubation, the cells were subjected to microscopic analysis, and the dynamics of GFP-RecN and HU-mCherry were tracked at 1 min intervals. GFP-RecN foci predominantly localized to nucleoid gaps as early as 30 min after release, and a subsequent recovery of HU-mCherry fluorescence intensity was observed at the same positions (Fig. 5*B* and Movie S1). These findings suggest a direct correlation between the localization of RecN and the recovery of nucleoid structure. In contrast, GFP-RecN^K35A^ foci remained at nucleoid gaps even after 60 min (Fig. 5*B* and Movie S2). The kinetics of cellular morphology for Δ*recN*/pBAD-GFP-RecN and Δ*recN*/pBAD-GFP-RecN^K35A^ cells revealed that the percentage of cells with fragmented nucleoids decreased in Δ*recN*/pBAD-GFP-RecN cells at 70 min after release from MMC exposure. In contrast, this percentage tended to increase in Δ*recN*/pBAD-RecN^K35A^ cells (Fig. 5*C*). These results demonstrate that RecN plays a pivotal role in restoring intact nucleoid organization by reactivating the HR reaction upon its recruitment to the site of damage in the nucleoid gap.

### Subcellular localization of RecA in response to DNA damage in the WT and Δ*recN* strains

Recent studies have revealed that the recruitment of RecN to the sites of DNA damage depends on the presence of RecA-ssDNA filaments (32, 33, 37, 43), indicating a potential correlation between RecA and nucleoid gap localization. To explore this hypothesis, we examined the subcellular localization of RecA upon induction of DNA damage using a plasmid (pRecA) expressing RecA from the T7 promoter under noninducing conditions. Previous studies have demonstrated that Δ*recA* cells carrying pRecA were fully functional in various assays (32, 48). Moreover, the amount of RecA in Δ*recA*/pRecA cells was comparable to that of chromosomally expressed *recA* in the presence of MMC, although it was expressed at relatively high levels in the absence of MMC (Fig. S3*A*).

To analyze the *in vivo* dynamics of RecA in response to DNA damage, we constructed a plasmid expressing a fluorescent protein–tagged RecA, connected by a G-S-T linker at its C terminus to mCherry (pRecA-mCherry). We found that Δ*recA* cells expressing RecA-mCherry exhibited higher MMC resistance than Δ*recA* cells carrying the empty plasmid vector, albeit showing increased sensitivity to high MMC concentrations (1.0 μg/ml) (Fig. S3*B*). This result is in line with previous studies indicating the partial functionality of the RecA-mCherry fusion protein (33, 49, 50). Therefore, we employed the RecA-mCherry fusion protein to monitor RecA dynamics in the presence of MMC (0.25 μg/ml), a concentration at which RecA-mCherry appears to function effectively.

**Figure 6:**
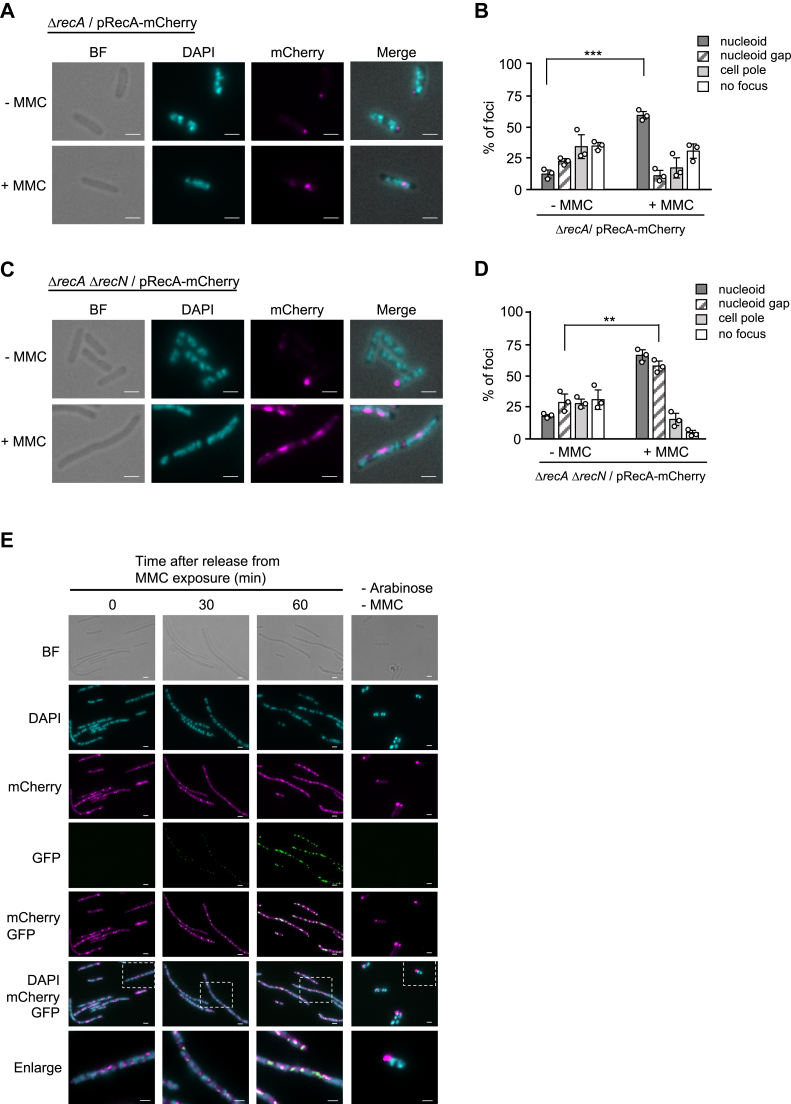
**Colocalization of GFP-RecN and RecA-mCherry in the nucleoid gap.***A*, Δ*recA*/pRecA-mCherry cells grown to early log phase were exposed to MMC (0.25 μg/ml) for 90 min. Cells were fixed and stained with DAPI, and analyzed by fluorescence microscopy. The panels show mCherry, DAPI, and merged mCherry/DAPI images of representative cells. Scale bar represents 2.0 μm. *B*, quantitative analysis of RecA-mCherry foci in (*A*). At least 100 cells were examined for each sample. The results represent the average of three independent measurements. Error bars indicate mean ± SD. *C*, Δ*recA* Δ*recN*/pRecA-mCherry cells were treated as in (*A*). Cells were analyzed by fluorescence microscopy. Scale bar represents 2.0 μm. *D*, quantitative analysis of RecA-mCherry foci in (*C*). At least 100 cells were examined for each sample. The results represent the average of three independent measurements. Error bars indicate mean ± SD. *E*, Δ*recA* Δ*recN* cells carrying both pRecA-mCherry and pBAD-GFP-RecN were exposed to MMC (0.25 μg/ml) for 90 min and then released into MMC-free medium with arabinose. Cells were fixed, stained with DAPI, and analyzed by fluorescence microscopy at the indicated time points. The panels show GFP, mCherry, DAPI, and GFP/mCherry-merged images, as well as BF images of representative cells after GFP-RecN induction. Scale bar represents 2.0 μm. *p* Values were calculated by unpaired Student's *t* test (*B* and *D*); ∗∗*p* < 0.01; ∗∗∗*p* < 0.001. BF, bright field; DAPI, 4,6-diamidino-2-phenylindole; MMC, mitomycin C.

To examine the subcellular localization of RecA, Δ*recA* cells expressing RecA-mCherry were treated with or without MMC for 90 min. In the absence of MMC, a subset of RecA-mCherry formed aggregated foci at the cell pole but did not form foci associated with the nucleoid (Fig. 6, *A* and *B*). However, upon exposure to MMC, the nucleoid structures remained intact, and RecA-mCherry foci localized to nucleoids (Fig. 6, *A* and *B*). This result supports the notion that MMC-induced RecA recruitment to nucleoids corresponds to the formation of RecA filaments at the sites of DNA damage. We next monitored the localization of RecA-mCherry in the Δ*recN* background during MMC exposure. When Δ*recA* Δ*recN*/pRecA-mCherry cells were treated with MMC, long filamentous cells with fragmented nucleoids were evident 90 min after MMC addition, indicating a functional RecA-mediated SOS response (Fig. 6*C*). In MMC-treated cells, various foci were observed, each with distinct fluorescence intensities. Notably, the foci with the high intensity were predominantly localized either on the nucleoids or in the nucleoid gaps (Fig. 6, *C* and *D*). These results suggest that the localization of RecA to the nucleoid gap is specific to MMC-treated Δ*recN* cells.

### RecN and RecA colocalized to nucleoid gaps in cells with the fragmented nucleoid structures

To determine whether RecA colocalizes with RecN in MMC-treated Δ*recN* cells, we examined the localization of RecA and RecN in MMC-treated Δ*recA* Δ*recN* cells harboring both pBAD-GFP-RecN and pRecA-mCherry. After treatment with MMC for 90 min in the absence of arabinose, cells were released into fresh MMC-free medium and further incubated for 60 min in the presence of arabinose to induce GFP-RecN. As expected, after MMC treatment, multiple RecA-mCherry foci were observed, but no GFP-RecN signal was detected (Fig. 6*E*; 0 min). Instead, GFP-RecN foci were observed at nucleoid gaps after the induction of GFP-RecN and completely colocalized with RecA-mCherry foci (Fig. 6*E*; 30 and 60 min). These results demonstrate that RecA recruits RecN to nucleoid gaps for HR-dependent DSB repair. We note that cells expressing both GFP-RecN and RecA-mCherry in the Δ*recA* Δ*recN* background did not show recovery of nucleoid aberrations throughout the experiments, although their nucleoid gap localizations were comparable to those in repair-proficient strains with fluorescent tags fused to either protein. This suggests that the addition of fluorescent tags to both proteins causes a defect in the HR pathway. Nevertheless, colocalization of GFP-RecN and RecA-mCherry was also observed on nucleoids when Δ*recA* Δ*recN* cells carrying both pSOS-GFP-RecN and pRecA-mCherry were treated with MMC (Fig. S4). Thus, it is likely that the dynamic behavior of RecA and RecN in response to MMC is not affected in Δ*recA* Δ*recN* cells expressing both GFP-RecN and RecA-mCherry. These results suggest that when the fluorescent tags were added to both proteins, they are proficient in recruiting to the site of DNA damage but fail to progress to subsequent steps of the HR pathway.

## Discussion

**Figure 7:**
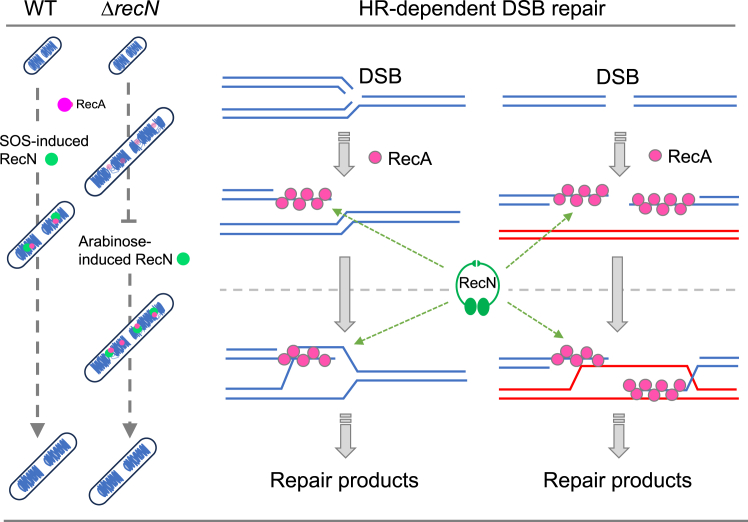
**A model for the role of RecN in the RecA-mediated HR pathway.** The treatment with MMC can result in the formation of both one-ended and two-ended DSBs. One-ended DSBs are primarily generated during DNA replication, whereas two-ended DSBs can occur because of the cleavage or removal of adducts. In *recN*-deficient strains, the processes following presynaptic stage are inhibited, leading to nucleoid decompaction around DSB sites and nucleoid fragmentation. Expression of RecN at this stage can resume the HR pathway by coordinating chromosome and RecA dynamics and subsequent restoration of nucleoid organization. Our findings suggest that RecA-mediated HR reactions need to be coordinated with chromosome dynamics, highlighting the essential role of RecN in this process. HR, homologous recombination; MMC, mitomycin C.

In this study, we focused on investigating the dynamics of RecN before and after perturbation of the nucleoid structure by inducing RecN from the *P*_*BAD*_ promoter at different time points. When like SOS-induced RecN, RecN expression was induced immediately after the initiation of MMC treatment, the nucleoid structure remained intact during DNA damage, and no fragmentation was observed. In this regard, it has been shown that RecN plays a role in preserving sister chromatid interactions following DNA damage (33, 34). This function may be particularly important during MMC treatment, as MMC-induced DNA damages can cause replication fork stalling and/or collapse, leading to the formation of DSBs (51, 52, 53, 54). Indeed, GFP-RecN foci were efficiently observed when cells were in early logarithmic phase, but not in stationary phase, suggesting that replication-dependent DSBs are generated in MMC-treated cells. Thus, rapid expression of RecN in response to damage plays an important role in maintaining nucleoid integrity during DSB repair. On the other hand, when RecN was induced in Δ*recN* cells after treatment with MMC, which caused the disruption of chromosome integrity, it restored normal nucleoid structures and promoted cell survival. Thus, the aberrant nucleoid structures observed in MMC-treated Δ*recN* cells do not represent irreversible chromosome disruption but rather an interruption of the RecA-mediated HR process. Our ﬁndings support the idea that the presynapsis and the synapsis in the HR pathway are separable in time and space, where SMC-like RecN plays structural and functional roles in facilitating a series of RecA-mediated reactions (Fig. 7).

How does RecN allow for the reconstruction of aberrant nucleoid structures? Interestingly, our imaging results revealed that when RecN was induced in MMC-treated Δ*recN* cells, it was predominantly localized to nucleoid gap regions, including the edges of the nucleoid. This is in contrast to SOS-induced RecN, which forms nucleoid-associated foci when rapidly expressed upon DNA damage (32, 33). Related to the nucleoid gap localization of RecN, we detected a weak HU-mCherry signal within these nucleoid gaps. The nucleoid in *E. coli* has a dynamic structure with individual supercoiled loops organized by nucleoid-associated proteins and topoisomerases as well as several scaffold proteins (46, 55). Thus, the nucleoid gap regions are not devoid of DNA but may correspond to regions of less compacted DNA where nucleoid organization has been locally disrupted as a result of DSBs. Taken together, we speculate that MMC-induced DSBs may lead to local disruption and/or relaxation of nucleoid organization in cells lacking cohesion function of RecN, resulting in the formation of nucleoid gaps. In support of this notion, the time-lapse imaging revealed that arabinose-induced RecN was recruited to a specific subset of nucleoid gaps in MMC-exposed Δ*recN* cells, and nucleoid structure was restored at the sites where RecN has localized. These results strongly suggest that the nucleoid gaps targeted by RecN contain DNA damage sites.

Our findings reveal that an ATPase-deficient RecN^K35A^ is able to localize to the nucleoid gap but fails to restore normal nucleoid structure. This implies that the ATP-binding and/or ATP-hydrolysis activity of RecN may not be necessary for its recruitment to the DSB site but is required for the subsequent restoration of normal nucleoid structure. Given that RecN has recently been shown to require ATP to induce DNA tethering and RecA-mediated strand exchange activity *in vitro* (35, 36), it is plausible that the ATP-dependent functions of RecN play a crucial role in restoring nucleoid integrity by modulating chromosome organization and RecA dynamics.

We demonstrated the localization of RecA to nucleoids and nucleoid gaps in MMC-treated Δ*recN* cells, where elongated and/or large RecA foci were predominantly observed. Our findings are consistent with a previous study (33), which reported the presence of elongated RecA filaments in MMC-treated Δ*recN* cells but not in WT cells. Moreover, it has been reported that RecA-ssDNA filaments can stretch to form long elongated RecA filaments and/or RecA bundles to facilitate pairing between DSBs and homologous donor strands located at distant sites (43, 56, 57). Consequently, it is plausible that MMC-treated cells, even if replication-dependent DSBs occur, maintain an apparently normal nucleoid structure through the RecN-dependent cohesion function. However, in the absence of RecN, the lack of sister chromatid interactions leads to a localized disruption of the nucleoid structure surrounding the DSB site. This disruption generates RecA-ssDNA structures within nucleoid gaps, potentially helping to maintain the cell in a stalled presynaptic phase by preventing unscheduled DNA degradation. We propose that arabinose-induced RecN is recruited to the sites of DSBs by RecA and stimulates RecA dynamics, even when chromosome integrity is compromised. This, in turn, resumes HR-dependent repair of DSBs (Fig. 7). Strikingly, a recent study by Chimthanawala *et al.* (37) demonstrated that RecN facilitates RecA filament dynamics, enabling long-distance homology searches. Moreover, *in vitro* study revealed that RecN binds to RecA-ssDNA filaments and captures a second dsDNA molecule in an ATP-dependent manner, facilitating RecA-mediated strand exchange for the repair of DSBs (36). These results suggest that RecN has distinct structural and functional roles in RecA-mediated DSB repair, potentially preserving sister chromatid interactions and stimulating RecA filament dynamics and strand exchange. However, the precise mechanisms underlying this process necessitate further investigation.

In conclusion, our work has provided insights into the dynamic behavior of nucleoid structures mediated by RecN during HR-dependent DSB repair. We have demonstrated that, even after chromosome integrity is compromised, RecN localizes to the nucleoid gaps alongside RecA, and can facilitate RecA-mediated DSB repair. These findings provide evidence for a two-stage model of the HR pathway; the presynaptic process, including RecA nucleoprotein filament formation, and subsequent synaptic processes in the HR pathway are separable in time and space. SMC-like RecN plays a crucial role in facilitating a series of RecA-mediated reactions by effectively coordinating both RecA and chromosome dynamics (Fig. 7). In eukaryotes, SMC family proteins have been found to localize to DSBs and promote HR-dependent repair, although the underlying mechanism remains unknown. Further investigations are warranted to explore the generalizability of the chromosome dynamics uncovered in our study to different biological systems. These future studies will shed light on the broader applicability and significance of our findings.

## Experimental procedures

### Strains and plasmids

The *E. coli* strains used in this study were derivatives of BW25113, except for MECS123 (Table 1). The WT strains and deletion mutants were obtained from the National BioResource Project. The strain carrying *hapA*-mCherry (MECS123) was generously provided by T. Katayama and S. Ozaki from Kyushu University. The WT strain and the *recN* deletion mutant were obtained from the National BioResource Project. Deletion alleles containing the kanamycin or tetracycline resistance gene were introduced into the BW25113 or MECS123 background through a lambda Red-based recombination method as described previously (58). The gene disruptions were confirmed by PCR using appropriate primers.

**Table 1:** List of strains used in this study

Strains	Background	Genotype	Source
ME9062	BW25113	Δ*(araD-araB)567,* Δ*lacZ4787(::rrnB-3), λ-, rph-1,* Δ*(rhaD-rhaB)568, hsdR514*	NBRP^a^
JW2669-KC	BW25113	Δ*(araD-araB)567*, Δ*lacZ4787(::rrnB-3)*, *λ-*, *rph-1*, Δ*(rhaD-rhaB)568*, *hsdR514* Δ*recA::Kan*^*R*^	NBRP^a^
JW5416-KC	BW25113	Δ*(araD-araB)567*, Δ*lacZ4787(::rrnB-3)*, *λ-*, *rph-1*, Δ*(rhaD-rhaB)568*, *hsdR514* Δ*recN::Kan*^*R*^	NBRP^a^
JW1852-KC	BW25113	Δ*(araD-araB)567*, Δ*lacZ4787(::rrnB-3)*, *λ-*, *rph-1*, Δ*(rhaD-rhaB)568*, *hsdR514* Δ*ruvC::Kan*^*R*^	NBRP^a^
SN001	BW25113	Δ*(araD-araB)567*, Δ*lacZ4787(::rrnB-3)*, *λ-*, *rph-1*, Δ*(rhaD-rhaB)568*, *hsdR514* Δ*recA::Kan*^*R*^ Δ*recN::Tet*^*R*^	This study
SN002	BW25113	Δ*(araD-araB)567*, Δ*lacZ4787(::rrnB-3)*, *λ-*, *rph-1*, Δ*(rhaD-rhaB)568*, *hsdR514* Δ*recN* Δ*ruvC::Kan*^*R*^	This study
MECS123	MG1655	*λ-*, *rph-1 hupA-mCherry*	T. Katayama, S. Ozaki
SN003	MG1655	*λ-*, *rph-1 hupA-mCherry* Δ*recN::Kan*^*R*^	This study

a National BioResource Project.

A *recN* fragment containing its native SOS promoter was cloned into pSTV28, generating pSOS-RecN. The arabinose-inducible pBAD-RecN (pTF271) was constructed as described previously (28, 32). To construct the plasmid expressing the Walker A mutant, *recN*^*K35A*^, site-directed mutagenesis by PCR was performed; two appropriate synthetic 30-mer oligonucleotides and pUC19-*recN* (WT) were used to change codon 35 of *recN* from AAA (Lys) to GCA (Ala). Subsequently, the WT *recN* allele was substituted with the *recN*^*K35A*^ allele to generate pSOS-RecN^K35A^ and pBAD-RecN^K35A^. RecN was tagged with an enhanced GFP cassette at the N terminus of RecN to generate pSOS-GFP-RecN and pBAD-GFP-RecN. The fragment containing the open reading frame of *recA* was cloned into pT7-7, generating pRecA. Using the In-Fusion kit (Takara Bio), a DNA fragment containing the linker sequence (G-S-T as an amino acid) and the open reading frame of mCherry was inserted between the penultimate codon and the stop codon of the *recA* gene in the pRecA plasmid, generating pRecA-mCherry. The structures of the recombinant plasmids were confirmed by DNA sequencing.

### Media and general methods

The standard methods for *E. coli* genetics and recombinant DNA techniques are described by Miller (59) and Sambrook *et al.* (60). In this study, cells were grown in LB medium containing 1% NaCl and supplemented with appropriate antibiotics, such as ampicillin (50 μg/ml), chloramphenicol (100 μg/ml), and kanamycin (30 μg/ml). The expression of a gene under the *P*_BAD_ promoter was achieved by supplementing the liquid and solid mediums with 0.05% and 0.2% arabinose, respectively. To assess sensitivity to MMC, 10-fold serial dilutions of the cultures were spotted onto LB plates containing MMC and arabinose, if necessary. The plates were then incubated overnight at 37 °C. All liquid assays were performed in LB medium containing 1.0 μg/ml MMC, unless otherwise stated.

### Cell survival assay

Cells were grown to early log phase in LB at 37 °C. They were then treated with MMC (1.0 μg/ml) and incubated for 90 min. After 90 min incubation, arabinose (0.05%) was added to induce *recN* expression, and the cells were further incubated for 60 min. At specific time intervals, aliquots were collected and spread onto LB plates with appropriate dilutions. After 20 h of incubation, colony counting was performed. Percent survival was calculated by comparing the number of colonies in treated samples relative to control samples (without MMC). The data presented are the mean values from at least three independent experiments (mean ± SEM).

### Western blot analysis

Exponentially growing cultures were treated with 1.0 μg/ml MMC or 0.05% arabinose. Aliquots were taken at the indicated times and centrifuged. Cells were resuspended in SDS denaturation buffer (40 mM Tris–HCl/pH 6.8, 7 M urea, 2 M thiourea, 0.3 M NaCl, 2% SDS, 0.1% bromophenol blue, 10% glycerol, and 2% 2-mercaptoethanol) and lysed by boiling. Samples were analyzed by SDS-PAGE as described previously (28). Western blot was performed using an anti-RecN antibody and the Western BLoT Ultra Sensitive HRP kit (Takara Bio). Anti-rabbit polyclonal antibody was produced against recombinant full-length RecN purified by chromatography (28).

### Fluorescence microscopy

Fluorescence microscopy was performed as described previously (28, 32). Exponentially growing cultures were treated with MMC in the presence or the absence of arabinose at 37 °C. Cells were harvested at the indicated times, fixed with ethanol, and stained with 1 μg/ml DAPI. The samples were then spread onto slide glass. Fluorescence microscopy was performed using either an Axioplan2 (Zeiss) or a BZ-X710 (Keyence) microscope, equipped with a 100× magnification oil-immersion objective. BZ-X Analyzer software (Keyence) was used for image processing. The number of nucleoids per cell and the localization of GFP-RecN foci were determined based on these visual criteria, and more than 100 individual cells were scored for each strain.

### Time-lapse observation

MECS Δ*recN*/pBAD-GFP-RecN cells were grown in LB medium at 37 °C until reaching the early log phase. MMC was added to the cultures and incubated for 90 min. After the 90 min incubation, arabinose (0.05%) was added to induce *recN* expression, and the cultures were further incubated for 30 min. Aliquots of the cell cultures were diluted and mounted on agarose pads containing M9 buffer and 1% arabinose. The MMC concentration was adjusted to 1.5 μg/ml to facilitate the detection of filamentous cells with fragmented nucleoids on the agar pad. Time-lapse images were taken every 1 min under an all-in-one fluorescence microscope (BZ-X710; Keyence) equipped with a time-lapse module (BZ-H4XT; Keyence).

## Data availability

All data are available in the main text or the supporting information.

## Supporting information

This article contains supporting information.

## Conflict of interest

The authors declare that they have no conflicts of interest with the contents of this article.
