# Supplementary material for: RecN spatially and temporally controls RecA-mediated repair of DNA double-strand breaks
Source: J Biol Chem. 2023 Nov 17;299(12):105466. doi: 10.1016/j.jbc.2023.105466 (PMC10714372; doi:10.1016/j.jbc.2023.105466)
Supplement: Supporting information [file mmc3.pdf]

Supporting Information

RecN spatially and temporally controls RecA-mediated repair of DNA double-strand breaks

Shunsuke Noda, Genki Akanuma, Kenji Keyamura, and Takashi Hishida

Included materials: Supporting Figure S1-S4

Supporting Figure S1

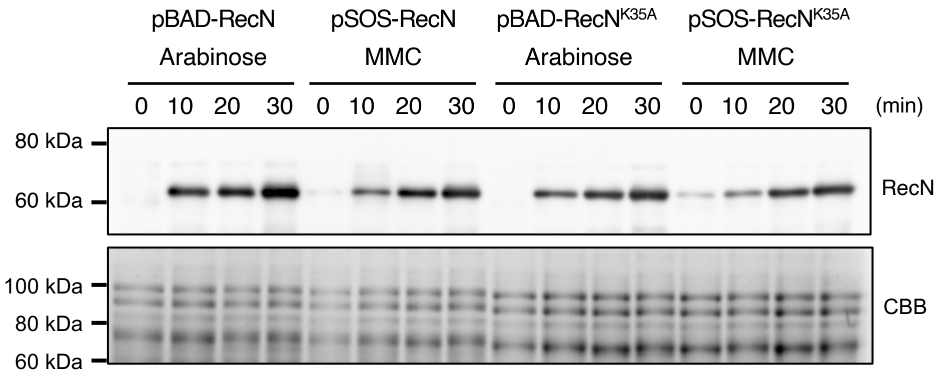

Figure S1. RecN protein levels upon induction of RecN using the P_{BAD}-promoter. Δ*recN* cells carrying pBAD-RecN were treated with 0.05% arabinose for 30 min. Δ*recN* cells carrying pSOS-RecN were treated with 1.0 µg/mL MMC for 30 min. Cells were harvested at the indicated time points. Crude cell extracts were resolved by SDS-PAGE for Western blot analysis with an anti-RecN antibody and Coomassie Brilliant Blue (CBB) staining. CBB staining served as a loading control for immunoblot. Protein molecular weight standards were used MagicMark™XP (Thermo Fisher).

Supporting Figure S2

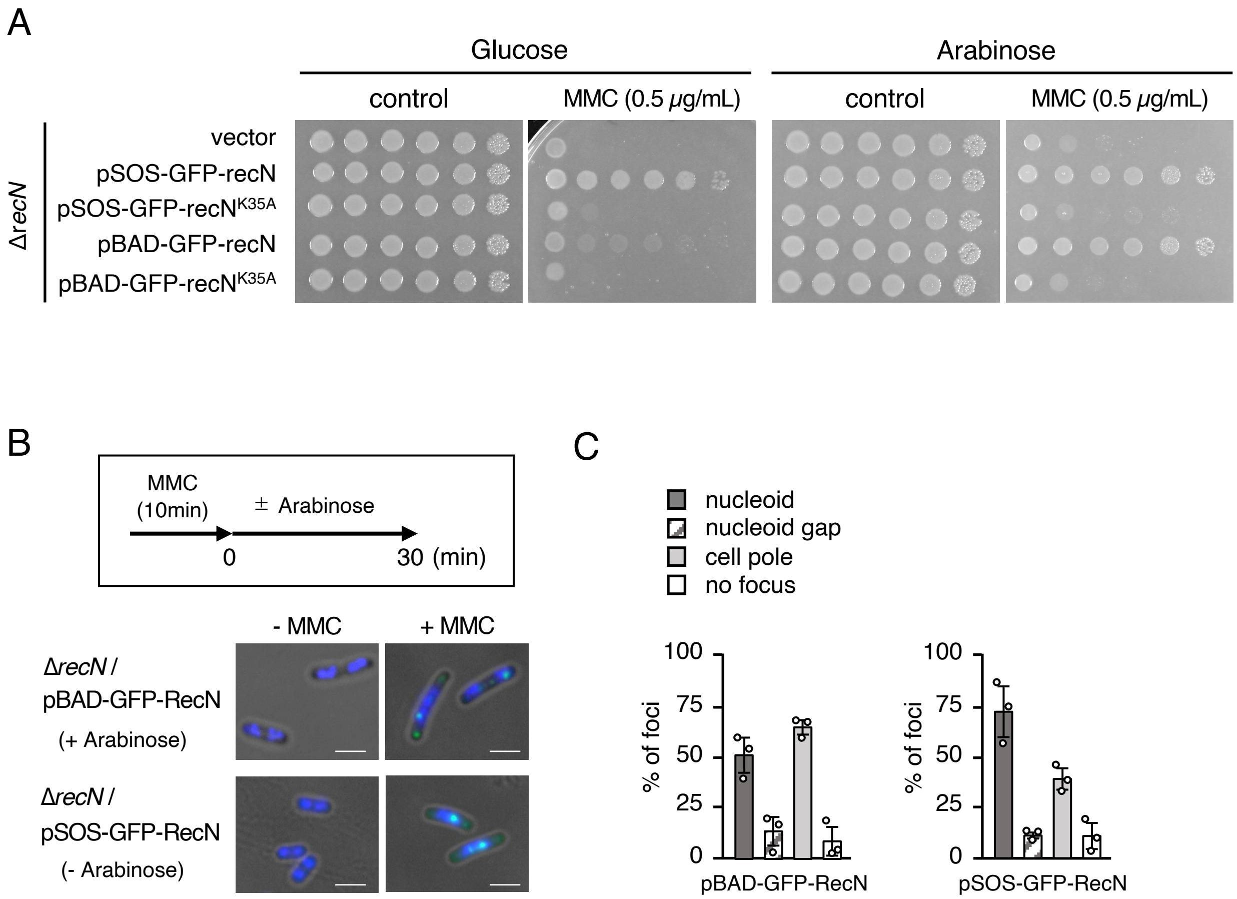

Figure S2. (**A**) Ten-fold serial dilutions of cell cultures were spotted onto LB_Cm plates with or without MMC in the presence of glucose or arabinose. The plates were incubated at 37°C overnight. (**B**) Nucleoid-associated localization of GFP-RecN. Δ*recN* cells carrying pBAD-GFP-RecN were exposed to MMC for 10 min, washed, and transferred to MMC-free medium. Arabinose was added at the beginning of the experiment. Cells were fixed and stained with DAPI and analyzed by fluorescence microscopy (*upper panels*). Δ*recN* cells carrying pSOS-GFP-RecN were treated similarly to the above except for the absence of arabinose (*lower panels*). The panels show merged GFP/DAPI/BF images of cells before and after MMC treatment. Nucleoids are visualized as a dark blue color. Scale bar: 2.5 μm. (**C**) Quantitative analysis of GFP-RecN foci in (**B**). At least 100 cells were examined for each sample. The results represent the average of three independent measurements (mean ± SD).

Supporting Figure S3

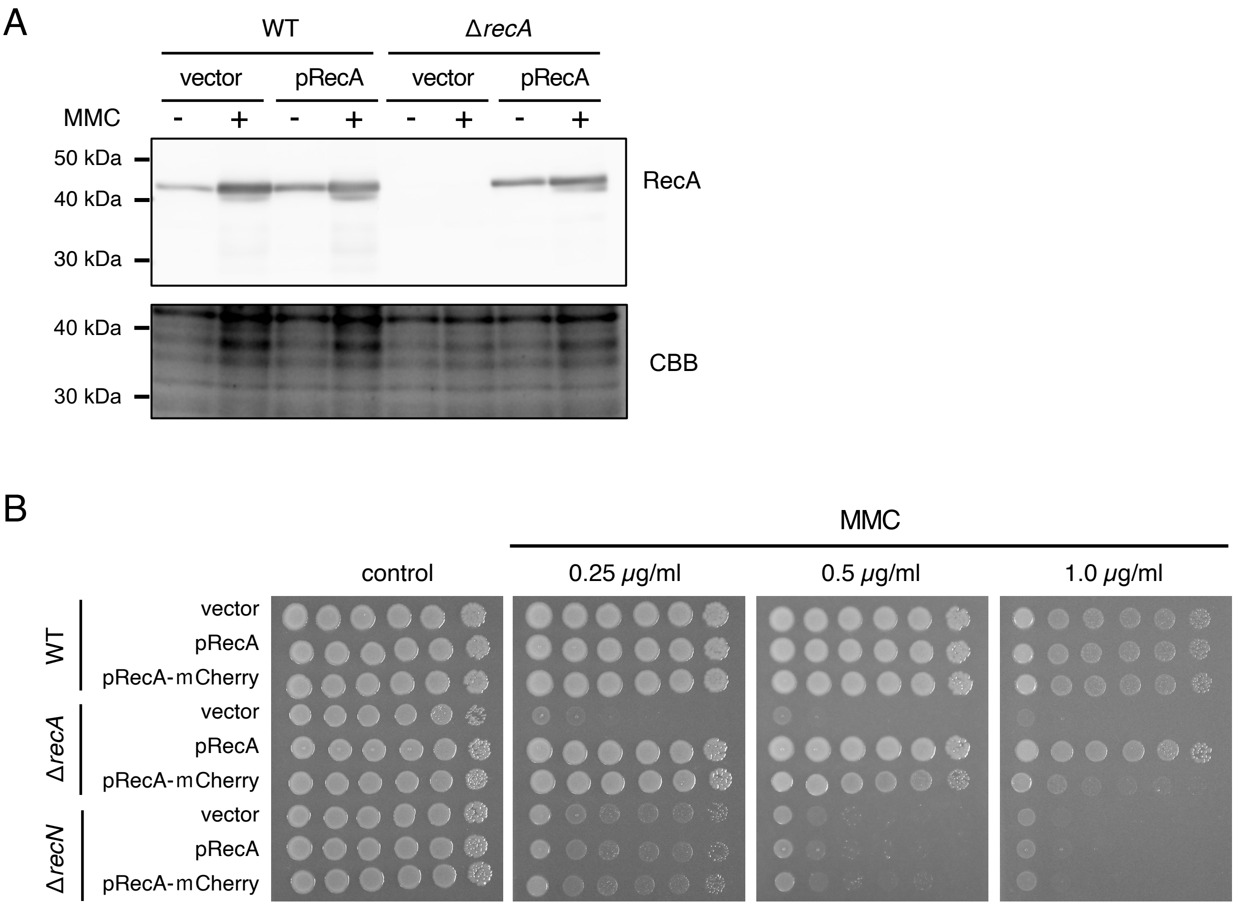

Figure S3. (**A**) RecA protein level in wild-type and Δ*recA*/pRecA cells in the presence or absence of MMC. Exponentially growing WT and Δ*recA* cells carrying either an empty vector or a pRecA were treated with 1.0 µg/mL MMC for 90 min. Cells were harvested before or after MMC treatment. Crude cell extracts were resolved by SDS-PAGE for Western blot analysis with an anti-RecN antibody and CBB staining. CBB staining served as a loading control for immunoblot. Protein molecular weight standards were used MagicMark^{TM}XP (Thermo Fisher). (**B**) Expression of RecA-mCherry confers partial MMC resistance to Δ*recA* cells. Ten-fold serial dilutions of the indicated cell cultures were spotted onto LB_Ap plates with or without MMC.

Supporting Figure S4

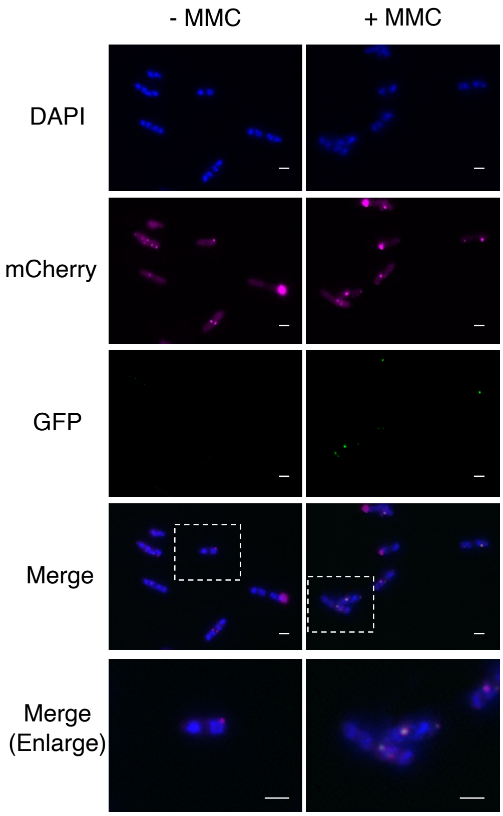

Figure S4. SOS-induced GFP-RecN colocalized with RecA-mCherry on the nucleoid. Δ*recA* Δ*recN* cells carrying both pRecA-mCherry and pSOS-GFP-RecN were grown to early log phase at 37°C in LB_Ap, Cm medium and treated with MMC for 10 min. Cells were then washed and released into MMC-free medium in the presence of arabinose. Cell cultures were incubated for an additional 30 min before microscopic analysis. The panels show mCherry, GFP, DAPI, and mCherry/GFP/DAPI-merged images. Scale bar: 2.0 µm.
